# The outcome of a surgical protocol based on ischemia overprotection in large and giant aneurysms of the anterior cerebral circulation

**DOI:** 10.1007/s10143-016-0721-z

**Published:** 2016-05-06

**Authors:** Hideaki Imai, Katsushige Watanabe, Takaaki Miyagishima, Yuhei Yoshimoto, Taichi Kin, Hirofumi Nakatomi, Nobuhito Saito

**Affiliations:** Department of Neurosurgery, Faculty of Medicine, The University of Tokyo Graduate School of Medicine, 7-3-1 Hongo, Bunkyo-ku, Tokyo, 113-8655 Japan; Department of Neurosurgery, Gunma University Graduate School of Medicine, Maebashi, Gunma Japan

**Keywords:** Bypass, Giant/large cerebral aneurysm, Internal carotid artery, Intracavernous aneurysm, Motor evoked potential, Perforating artery

## Abstract

Aiming to define the optimal treatment of large and giant aneurysms (LGAs) in the anterior circulation, we present our surgical protocol and patient outcome. A series of 42 patients with intracavernous LGAs (*n* = 16), paraclinoid (C2) LGAs (*n* = 17), and peripheral (middle cerebral artery—MCA or anterior cerebral artery—ACA) LGAs (*n* = 9) were treated after bypass under motor evoked potential (MEP) monitoring. Preoperatively, three categories of ischemic tolerance during internal carotid artery (ICA) occlusion were defined on conventional angiography: *optimal*, *suboptimal*, and *insufficient* collaterals. Accordingly, three types of bypass: low flow (LFB), middle flow (MFB) and high flow (HFB) were applied for the cases with *optimal, suboptimal*, and *insufficient* collaterals, respectively. Outcome was evaluated by the Glasgow Outcome Scale (GOS). All patients had excellent GOS score except one, who suffered a major ischemic stroke immediately after surgery for a paraclinoid lesion. Forty-one patients were followed up for 87.1 ± 40.1 months (range 13–144 months). Intracavernous LGAs were all treated by proximal occlusion with bypass surgery. Of paraclinoid LGA patients, 15 patients had direct clipping under suction decompression and other 2 patients with recurrent aneurysms had ICA (C2) proximal clipping with HFB. MEP monitoring guided for temporary clipping time and clip repositioning, observing significant MEP changes for up to 6 min duration. Of 9 peripheral LGAs patients 7 MCA LGAs had reconstructive clipping (*n* = 4) or trapping (*n* = 3) with bypass including LFB in 3 cases, MFB in 1 and HFB in 1. Two ACA LGAs had clipping (*n* = 1) or trapping (*n* = 1) with A3-A3 bypass. The applied protocol provided excellent results in intracavernous, paraclinoid, and peripheral thrombosed LGAs of the anterior circulation.

## Introduction

Despite the advancement and refinement of microsurgical and endovascular techniques, the treatment of giant intracranial aneurysms is still a challenging task [2, 6, 10, 15, 22, 24, 26, 32], even for experienced neurovascular surgeons. The promising outcomes achieved by endovascular therapy for small aneurysms nevertheless remain unconfirmed for giant aneurysms; larger case series report significant rates of recanalization and low percentages of complete closure [3, 7–9, 11, 17], and the most recent developments of endovascular techniques did not provide definite efficient alternatives for treatment to *open* surgery.

Generally, *open* surgical treatment of these aneurysms excludes the possibility to apply traditional surgical clipping techniques because they are often large and have complex shape, sclerosis of the neck accompanied with thrombosis and direct involvement of parent, collateral and perforating vessels. Since the surgical technique of clipping often includes volume reduction of the aneurysm by aspiration and/or thrombectomy to collapse it, at least temporary trapping is required.

The unclippable intracavernous aneurysms may require proximal carotid ligation [5]. However, ischemic damage during and after the procedure is the most significant risk during this treatment. To protect from eventual ischemic damage during the procedure, bypass surgery of different types was developed to compensate for hypoperfusion of the related brain area and adequate assessment for its indications is needed preoperatively. Careful selection of the donor-recipient site, grafting vessel type and size, and bypass technique has to be adjusted to an eventual flow deficit that might be induced with the treatment and after it [25]. Therefore, preoperative evaluation of ischemic tolerance to occlusion, in spite of all controversies on the existing methods, remains essential for management success in these lesions.

Within the same context, another remaining obstacle is the preservation of the terminal perforating arteries arising from the aneurysm and parent vessel portions that have to be occluded at surgery, with no established management strategy yet for this problem [21]. Therefore, we applied intraoperative motor evoked potential (MEP) monitoring to all patients in this study to see whether the function of the corticospinal tract is maintained during and after parent artery occlusion. The MEP monitoring is the most sensitive and reliable method to evaluate neurological function and alert surgeons of inadequate regional cerebral blood flow (CBF) during the surgical procedure [28].

Combining preoperative ischemia tolerance evaluation with intraoperative monitoring, since 2001 we decided to analyze the effectiveness of a strategy aiming to avoid the possibility of ischemic complications by *overprotective* bypass choice and MEP guided intraoperative flow control. The present study summarizes the results of this strategy with its related outcome in our institution in a consecutive case series of complex large and giant aneurysms (LGAs).

## Patients and Methods

A series of 42 patients (male/female, 13/29; age range, 32–74 years; average ± standard deviation [SD], 59.0 ± 9.9 years) with complex LGAs in the anterior circulation, treated surgically by the senior author (personal series of N.S.) between 2001 and 2013, were enrolled in this study. LGAs were divided into three categories according to the involved segment of the internal carotid artery (ICA) system: (1) intracavernous LGAs (16 cases) (male/female, 6/10; age range, 46–74 years; average ± SD, 58.8 ± 9.7 years), (2) paraclinoid LGAs (17 cases) (male/female, 3/14; age range, 45–73 years; average ± SD, 59.6 ± 8.7 years), and (3) peripheral-anterior cerebral artery (ACA) and middle cerebral artery (MCA)-LGAs (2 ACA and 7 MCA aneurysms) (male/female, 4/5; age range, 32–68 years; average ± SD, 52.6 ± 13.4 years).

### Angiographic assessment (balloon occlusion test)

Under systemic heparinization with a target-activated clotting time of more than 250 s, a 5 F sheath introducer and 4 F sheath introducer were placed in the right and left femoral artery, respectively. A 5.2 F double-lumen balloon catheter (Selecon MP catheter II; Terumo Clinical Supply, Japan) was positioned in the cervical portion beyond the carotid bifurcation into the ipsilateral ICA carrying the LGA. Immediately after occlusion of the ICA with inflation of the balloon, a total 7 mL of a contrast medium (Iohexol, 300 mg/mL) was injected at a rate of 3.5 ml/s through an 4 F angiographic catheter (Medikit angiography catheter; Medikit Co Ltd, Japan) that was placed in the contralateral internal carotid artery. Digital subtraction angiography was performed in an anterior–posterior view from the time of injection with the frame rate of 2 fps until all contrast media disappeared from cerebral circulation. Patients who developed any neurological symptom or sign such as consciousness disturbance, hemiparesis, aphasia, visual field defect or other under balloon occlusion test (BOT) were considered intolerant. Patients who did not develop neurological symptoms or deficit during the 15-min occlusion were considered tolerant. During the BOT, a mean stump pressure as well as physiological monitoring was recorded to confirm its safety with careful observation for clinical manifestations.

### Categorization of ischemic tolerance based on angiographic findings

**Fig. 1:**
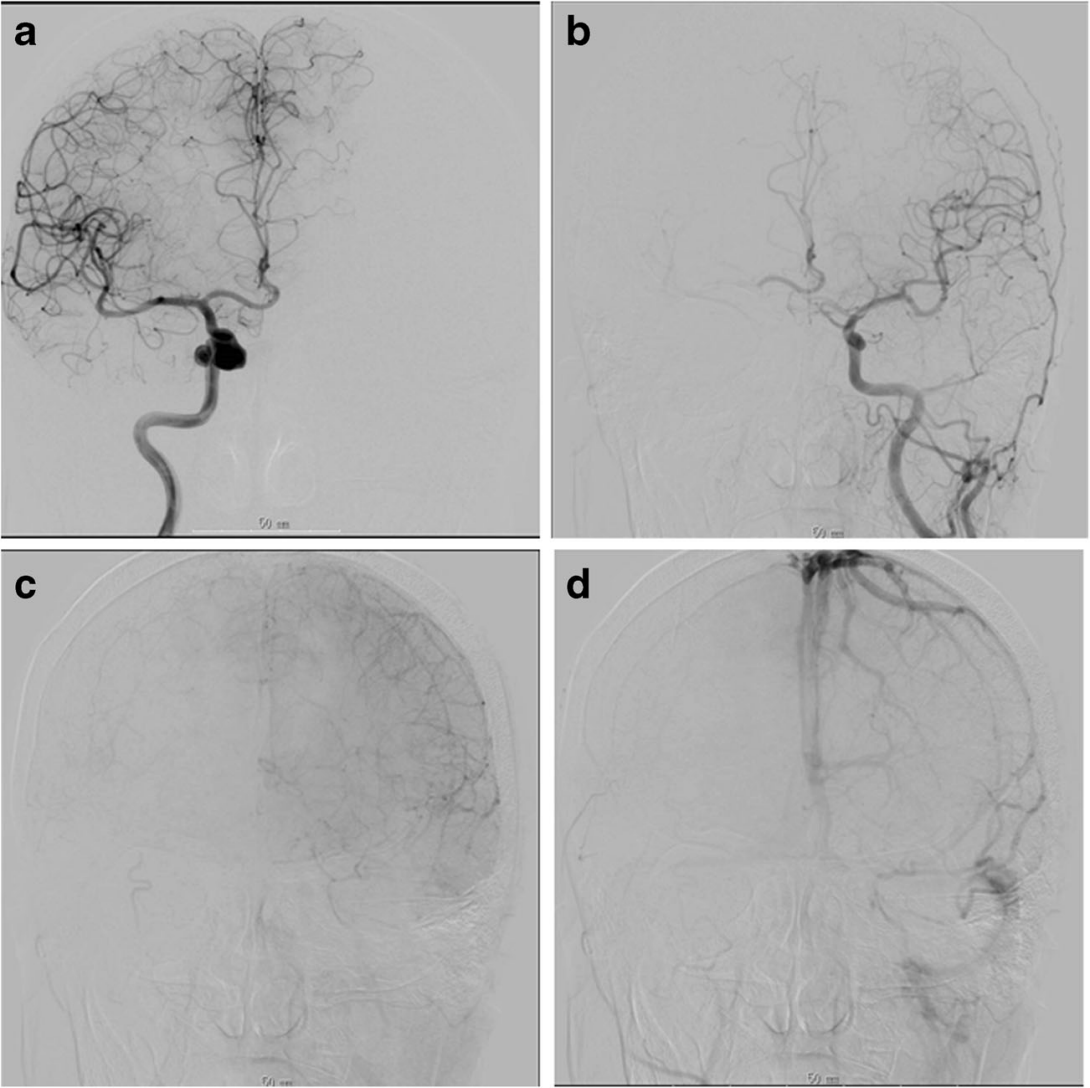
A woman with a large cavernous sinus aneurysm. **a** Preoperative right internal carotid angiogram, anteroposterior view, showing a large and irregularly shaped aneurysm in the cavernous sinus portion. **b**–**d** Left internal carotid angiograms with Matas test showing the insufficient collateral flow of contralateral hemisphere through the exposure from early arterial to venous phase

**Fig. 2:**
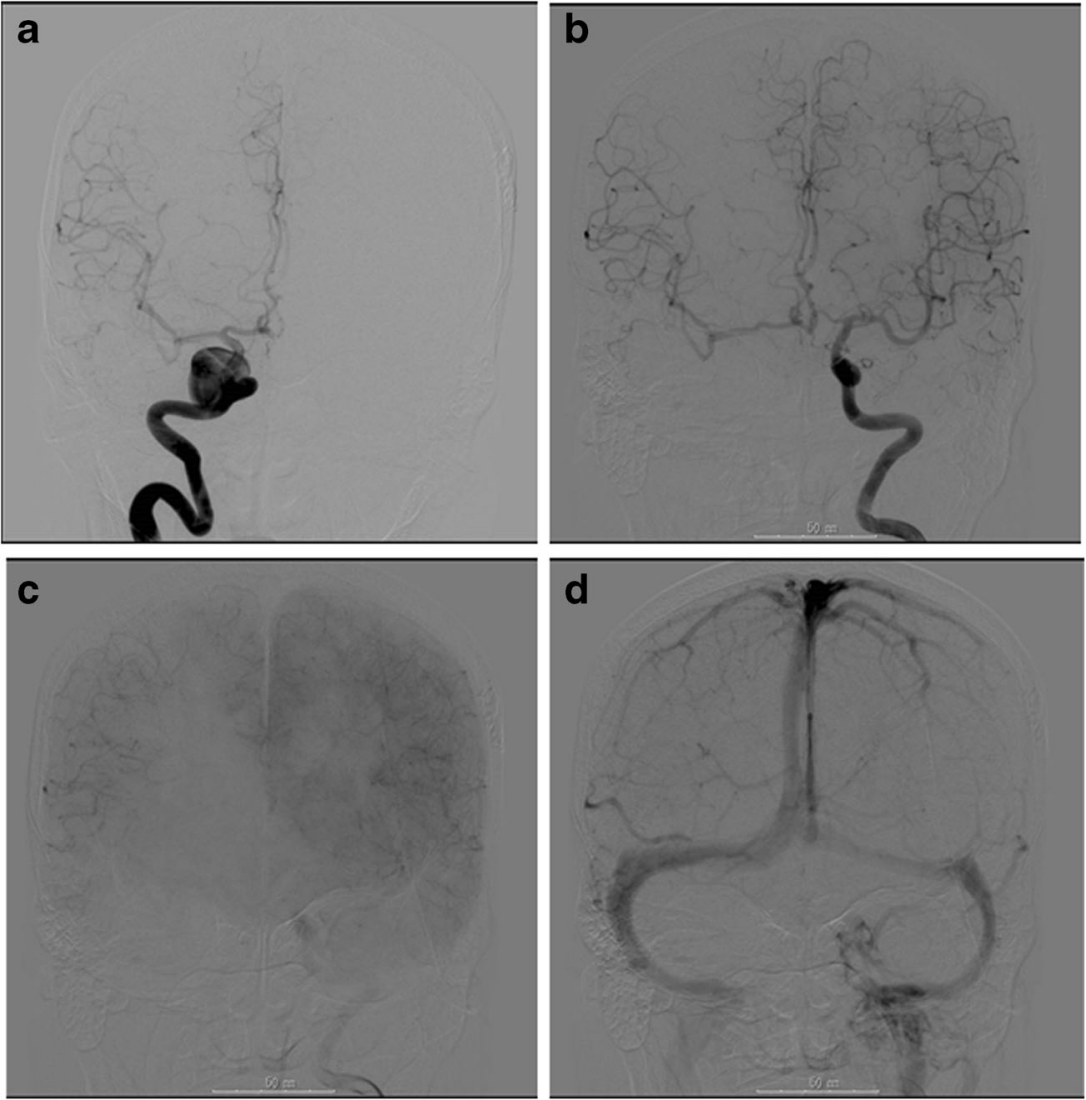
A woman with a giant cavernous sinus aneurysm. **a** Preoperative right internal carotid angiogram, anteroposterior view, showing a giant and round shaped aneurysm in the cavernous sinus portion. **b**–**d** Left internal carotid angiograms with Matas test showing the sufficient collateral flow of contralateral hemisphere from early arterial to venous phase. There is no apparent laterality of the amount and time shift of venous phase flow

**Fig. 3:**
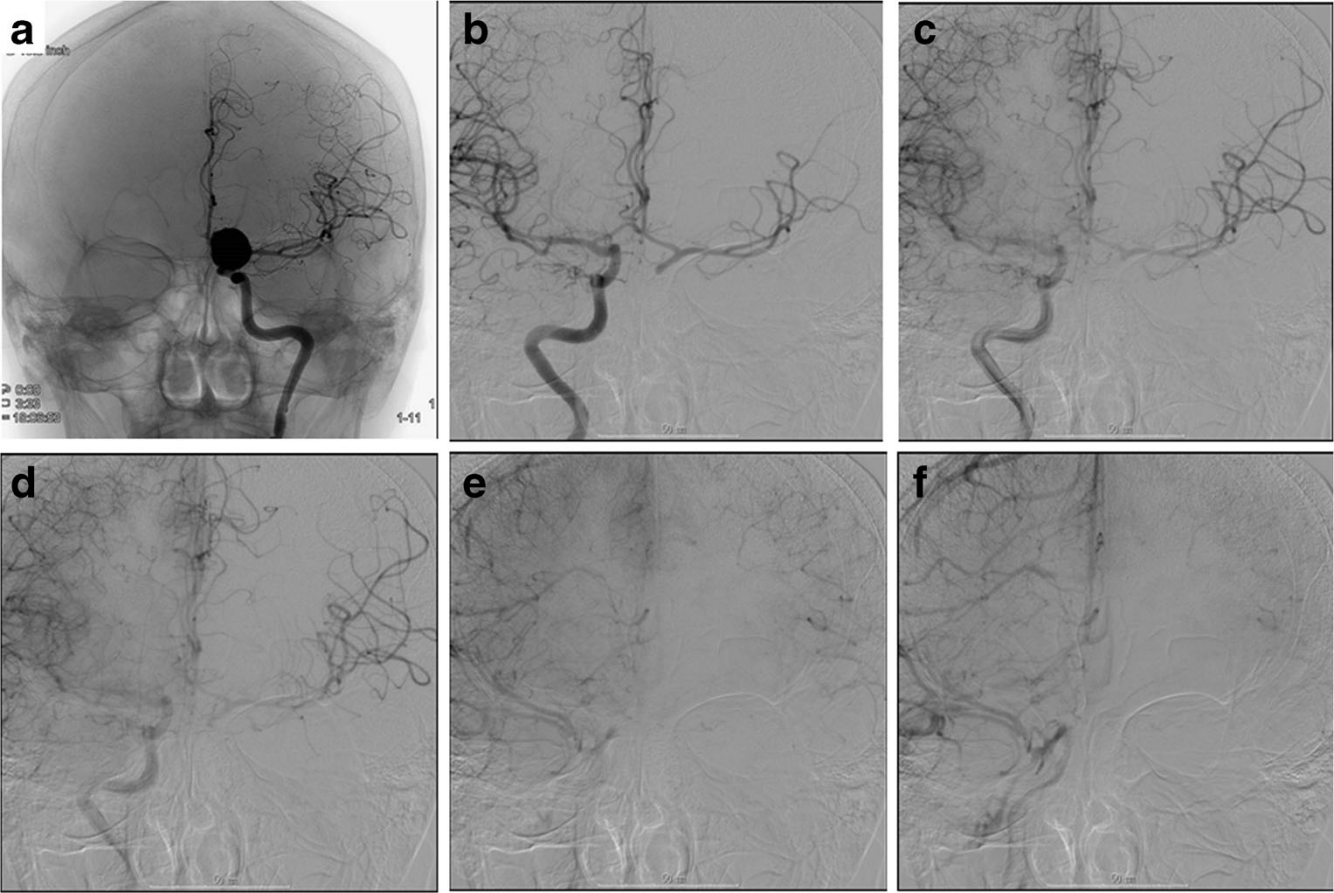
A woman with a giant paraclinoid aneurysm. **a** Preoperative left internal carotid angiogram, anteroposterior view, showing a giant and round-shaped aneurysm in the paraclinoid region. **b**–**f** Right internal carotid angiograms with Matas test showing the relatively good collateral flow of contralateral hemisphere in early arterial phase, with time shift of more than 2 s, best noticeable in the venous phase of the injected side (**d** and **e**)

As a general strategy based on the uncertainties of preoperative ischemia tolerance testing and the risks associated with intraoperative flow interruption in the ICA and its branches and perforators, we applied an *overprotective* bypass collateral supply principle in all cases. It consisted of establishing a bigger than the required bypass flow supply to the carotid territory harboring an intracavernous and paraclinoid aneurysm, based on the angiographic evaluation and preoperative clinical BOT. The preoperative angiographic assessment (including Matas and Alcock tests) was qualitative, dividing efficiency of collaterals at cross-compression of the carotid system of interest during contralateral carotid injection into *insufficient*, *suboptimal*, and *optimal* by the amount and timing of flow in the territory of the occluded carotid system. The *insufficient* category was obvious in case preoperative angiographic assessment with Matas test showed no adequate collateral flow to the contralateral side from arterial until venous filling phase of ipsilateral hemisphere (Fig. 1). In case there was sufficient collateral flow during the arterial phase on angiography under the Matas test, the timing of the venous phase on the compressed side was evaluated. The *suboptimal* category was differentiated from the *optimal* (Fig. 2) as 2 or more seconds delay of the venous phase regardless of the adequate contrast distribution to all peripheral territories and degree of opacification in the occluded carotid system (Fig. 3). Based on the established ischemia tolerance according to the protocol, we used three types of bypasses for flow preservation and augmentation according to the expected amount of flow they provide in descending order: the *high flow* bypass (HFB) for the *insufficient* category, the *middle flow* bypass (MFB) for the *suboptimal* category using interposed venous grafts from the superficial temporal artery (STA) trunk to an intracranial artery, and the *low-flow* bypass (LFB) for the *optimal* category that was represented by the STA-MCA or the A3-A3 anastomosis. MEPs were used for intraoperative evaluation of perforating artery occlusion tolerance.

### Intracavernous LGAs

The goal in this subgroup was to optimize a proximal carotid occlusion. Tolerance to ischemia was estimated preoperatively by angiographic evaluation complemented in part of the cases with clinical BOT (Table 1). Additionally, under MEP monitoring during surgery, provisional ICA occlusion for 30 min was applied to evaluate whether the indications of ischemic changes (amplitude decrease by 50 % or more) appeared. This was to reassure that ICA occlusion at the cervical portion under the hemodynamic conditions at the time of surgery should not have any adverse effect.

**Table 1:** Intracavernous LGA cases

Case No.	Age/Sex	Angiographic assessment	BOT	Bypass graft	MEP	Symptom (cranial nerve)	Post-op	Patency	GOS
1	56/M	Optimal	Negative	STA	No change	III, IV, V, VI	No change	Yes	GR
2	50/M	Optimal	Negative	STA	No change	VI	Improved	Yes	GR
3	58/F	Insufficient	N/A	RA	No change	V (pain), VI	Improved	Yes	GR
4	63/F	Insufficient	N/A	RA	No change	III, IV, V (pain), VI	Improved	Yes	GR
5	46/F	Suboptimal	N/A	Short SV	No change	VI	Improved	No	GR
6	53/F	Insufficient	N/A	RA	No change	III, V (pain), VI	Improved	Yes	GR
7	67/F	Insufficient	N/A	RA	No change	V (pain)	Improved	Yes	GR
8	74/F	Insufficient	Negative	RA	No change	III, IV, V (pain), VI	Improved	Yes	GR
9	57/M	Insufficient	Negative	RA	No change	II	No change	Yes	GR
10	62/M	Insufficient	Positive	RA	No change	V, VI	Improved	Yes	GR
11	62/F	Insufficient	Positive	SV	No change	No	No change	Yes	GR
12	70/M	Insufficient	Positive	RA	No change	III	No change	No	GR
13	62/F	Optimal	Negative	STA	No change	VI	No change	Yes	GR
14	55/M	Optimal	Negative	STA	No change	XII	No change	Yes	GR
15	70/F	Optimal	Negative	STA	No change	III, IV, VI	No change	Yes	GR
16	69/F	Insufficient	Positive	RA + STA	No change	Headache, dizziness	Improved	Yes	GR

*BOT* Balloon occlusion test, *GOS* Glasgow outcome scale, *GR* Good recovery, *LGA* Large and giant aneurysm, *MEP* Motor evoked potential, *RA* Radial artery, *STA* Superficial temporal artery, *SV* Saphenous vein

All patients who did not pass the BOT test or had *insufficient* angiographic collaterals (Fig. 1) had HFB, those who passed the BOT and had collaterals, however estimated as *suboptimal*, had MFB, and those with passed BOT and had adequate collateralization (*optimal*) (Fig. 2) had LFB.

### Paraclinoid LGAs

The same principles of angiographic evaluation were applied as in the previous group: the patients with expected trapping or proximal clipping that did not pass the BOT or had *insufficient* collateralization had HFB, those with preservable ICA (aneurysm clipping) but with *suboptimal* collaterals (Fig. 3) distal to the lesion had MFB, and those with preservable ICA and adequate collaterals had LFB. In four cases the performance of LFB was considered unnecessary due to the absence of STA donor with significant blood flow or the absence of appropriate infra-sylvian recipient, as the bypass should not interfere with the main part of the intracranial work through the Sylvian fissure.

Surgical technique in this subgroup depended on the morphological characteristics of the lesion—location, relation to adjacent neural and vascular structures, and the ischemia tolerance to parent vessel occlusion. Ischemia tolerance was required for aneurysm dissection, volume reduction and clipping, as we gave emphasis to the critical for ischemia points in the surgical plan. When dissection was considered to carry risks of inadequate control over the parent vessel, important branches and perforators, the suction-decompression technique (SDT) was applied, delaying as much as possible intentional rupture and thrombectomy. Of special interest was the relationship between aneurysmal neck and anterior choroidal/posterior communicating arteries, whose temporary obliteration was potentially hazardous for ischemia. MEP monitoring was applied through the whole intracranial part of the procedures. Temporary clipping of the parent vessel was applied for periods not exceeding 10 min under the provision that MEP amplitude has not decreased below 50 % during these periods. Clip repositioning and gradual achievement of the optimal clipping pattern, often on multiple steps, minimized occlusion time and allowed preservation of parent vessels and related branches and perforators.

### Peripheral (MCA and ACA) LGAs

MCA LGAs were treated under the same principles as those of paraclinoid location regarding ischemia protection, bypass choice, and intraoperative MEP monitoring. However, for ACA LGAs, we used an A3-A3 bypass instead of STA-MCA anastomosis.

### MEP monitoring

MEP monitoring was set according to the methods we have previously reported [31]. Under general anesthesia with remifentanil, propofol, and/or sevoflurane in oxygen, the initially given muscular relaxants were discontinued. Stimulation electrodes (cross-recessed head tapping screws), placed in the outer table of the skull over C3 and C4 (International 10–20 System), at least 2 cm away of the surgical incision, were connected to the combined stimulating and recording device (MEB-4300; Nihon Kohden, Inc., Tokyo, Japan). The MEPs were elicited by transcranial electrical stimulation with a train of five square-wave pulses (duration 400 μs, intensity 200 mA, frequency 500 Hz) delivered between the two screw electrodes, recording electromyographic responses from abductor pollicis brevis and tibialis anterior muscles bilaterally, filtering them between 100 and 1000 Hz. The threshold current intensity needed to evoke motor responses from target muscles was monitored throughout the operation, as indicated for threshold-level transcranial electrical stimulation [31].

### Surgical procedure

**Fig. 4:**
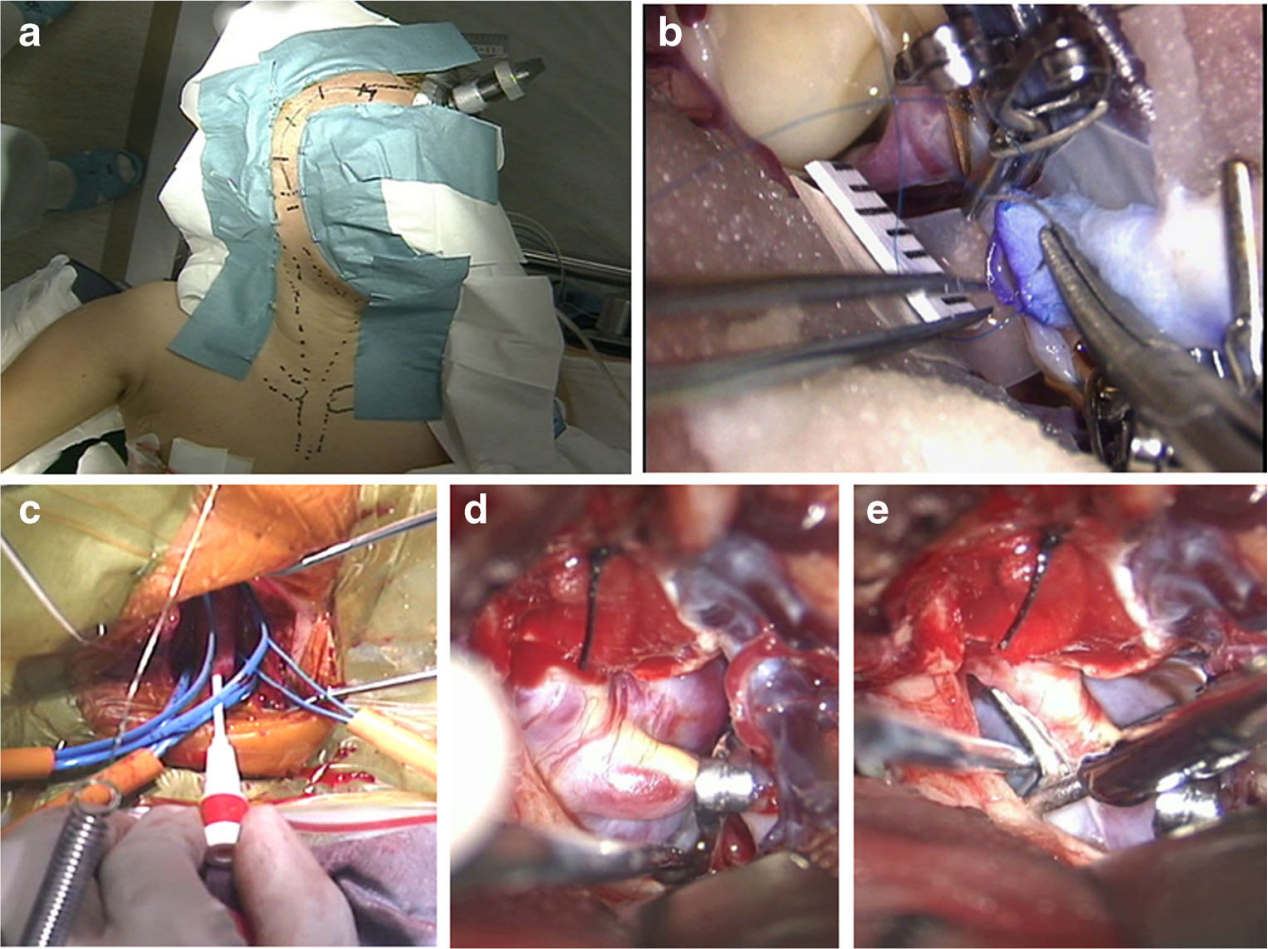
Short saphenous vein-M2 anastomosis and suction-decompression technique (SDT). **a** General surgical position and preparation for a complex LGA of the anterior circulation. **b** Surgical view of an end-to-side short saphenous vein-M2 anastomosis showing the running suture through the vessels walls with 8-0 nylon during temporary occlusion of M2. **c** A procedure for the retrograde SDT showing the preparation at the neck. 4 Fr angiocatheter was inserted via the common carotid artery into the ICA. Vascular tapes were set for the common carotid artery, ICA and external carotid artery for temporary occlusion. **d** Microsurgical view of the giant paraclinoid aneurysm after placing a temporary clip on the intracranial ICA distal to the aneurysm neck. The aneurysm still appears to be tense at this stage. **e** Intra-aneurysmal pressure under control by retrograde SDT, allowing the fenestrated clip to be applied. Clipping occluded the neck orifice and reconstructed the original arterial lumen

A standard pterional craniotomy was routinely used for all cases except for A2 LGAs cases. The frontotemporal and anterolateral cervical areas were prepared (Fig. 4a) and draped as a single surgical field. Ipsilateral to the approach arm or leg were also prepared and draped in the cases they were needed for harvesting donor vessels. Craniotomy, neck dissection, and donor vessel harvesting were usually performed simultaneously by two surgical teams.

In cases of intracavernous LGAs, the cervical ICA was exposed for proximal parent vessel control. Proximal ICA occlusion was preceded by the bypass procedure. For HFB, we applied the technique described by Houkin et al. [12] but we used a large vascular trocar to tunnel the graft between the cervical and the cranial incisions to minimize graft compression by soft tissues. The distal anastomosis was performed first in an end-to-side fashion to one of the M2 segments with 8–0 or 9–0 monofilament nylon sutures. To preserve distal collaterals, the proximal anastomosis was performed in an end-to-side manner to the external carotid artery using 7–0 monofilament nylon sutures.

In paraclinoid LGA cases, direct clipping was applied under proximal control of the parent vessel with retrograde SDT [1, 19, 20]. Initially, the cervical ICA was exposed and bypasses were performed according to the angiographic evaluation and BOT results. In MFB, the short saphenous vein graft was connected to the M2 segment performing first an end-to-side distal anastomosis with 8–0 or 9–0 monofilament nylon sutures (Fig. 4b). When required, to preserve perfusion via the distal collaterals, the proximal anastomosis was performed in an end-to-side manner to the STA using 8–0 monofilament nylon sutures.

For SDT, an angiographic catheter sheath was introduced into the cervical ICA after temporary proximal clamping (Fig. 4c) and the site distal to the aneurismal neck on ICA for temporary clipping was also exposed (Fig. 4d). When the trapping clip was distal to the posterior communicating artery, the intra-aneurysmal pressure might remain high, not allowing space for safe aneurysm dissection. In these cases, retrograde SDT produced aneurysm collapse and provided the surgeon with enough space to finalize dissection and clipping (Fig. 4e). When aneurysm obliteration could not be completed in a single step, this technique was applied repeatedly, moving each time the clip closer to the aneurysmal neck. These steps were sequenced at 5–10 min intervals until complete exclusion of the aneurysm from the circulation was achieved. Aspirated blood was usually collected in blood bags for possible re-transfusion.

Two patients had aneurysms that enlarged after previous incomplete coil embolization. They were treated by clipping with parent artery trapping and dome dissection to enable coil removal, without resorting to bypass surgery. Another two cases of recurrent giant aneurysms, one *de novo* and one with adjacent re-growth several years after being treated by direct clipping, were managed by ICA proximal occlusion—C2 clipping with HFB.

In cases of A2 LGAs, an interhemispheric approach was used to perform initially an A3-A3 bypass, then to expose the anterior communicating artery complex under temporary occlusion of A1 segments, and finally the aneurysm had neck clipping or trapping.

In thrombosed MCA aneurysms, the appropriate bypass was performed, and then the relevant branches of the MCA were trapped. After thrombectomy and dissection, the aneurysms were permanently trapped or clipped. MEPs were used through the whole period of intracranial work and significant amplitude decreases were managed by clip repositioning.

### Follow-up

All patients were followed up at regular intervals after discharge on outpatient visits (at least once a year) and evaluated by magnetic resonance (MR) imaging and/or computed tomography (CT) angiography as required.

This management protocol has been approved by the internal review board of the University of Tokyo Hospital, and written informed consent was obtained in all cases prior to participation in this study.

## Results

The mean clinical follow-up period for these 42 patients was 87.1 ± 40.1 months (range 13–144 months).

### Intracavernous LGA cases

**Fig. 5:**
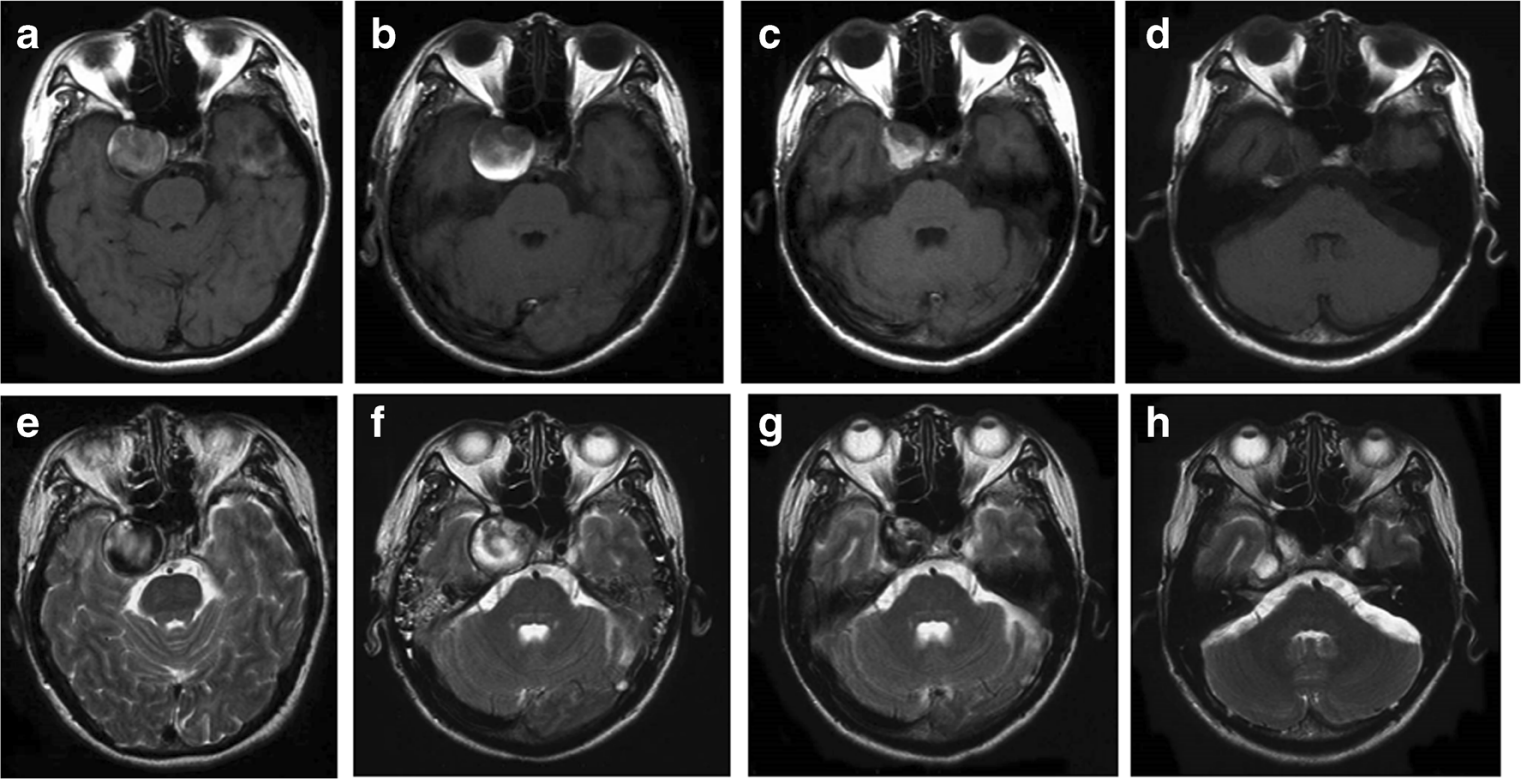
Series of MR imaging findings of a right intracavernous aneurysm before and after surgery; T1-weighted (**a**–**d**) and T2-weighted images (**e**–**h**). Preoperative MR images revealed a giant flow void and mass effect in the right cavernous portion (**a**, **e**). Notably, 1 day after surgical treatment T1-weighted (**b**) and T2-weighted images (**f**) showed high intensity inside the aneurysm, revealing the beginning of intra-aneurysmal thrombosis. Four months after treatment, the aneurysm remarkably shrank (**c**, **g**). Eighteen months after treatment the giant aneurysm almost disappeared (**d**, **h**)

**Fig. 6:**
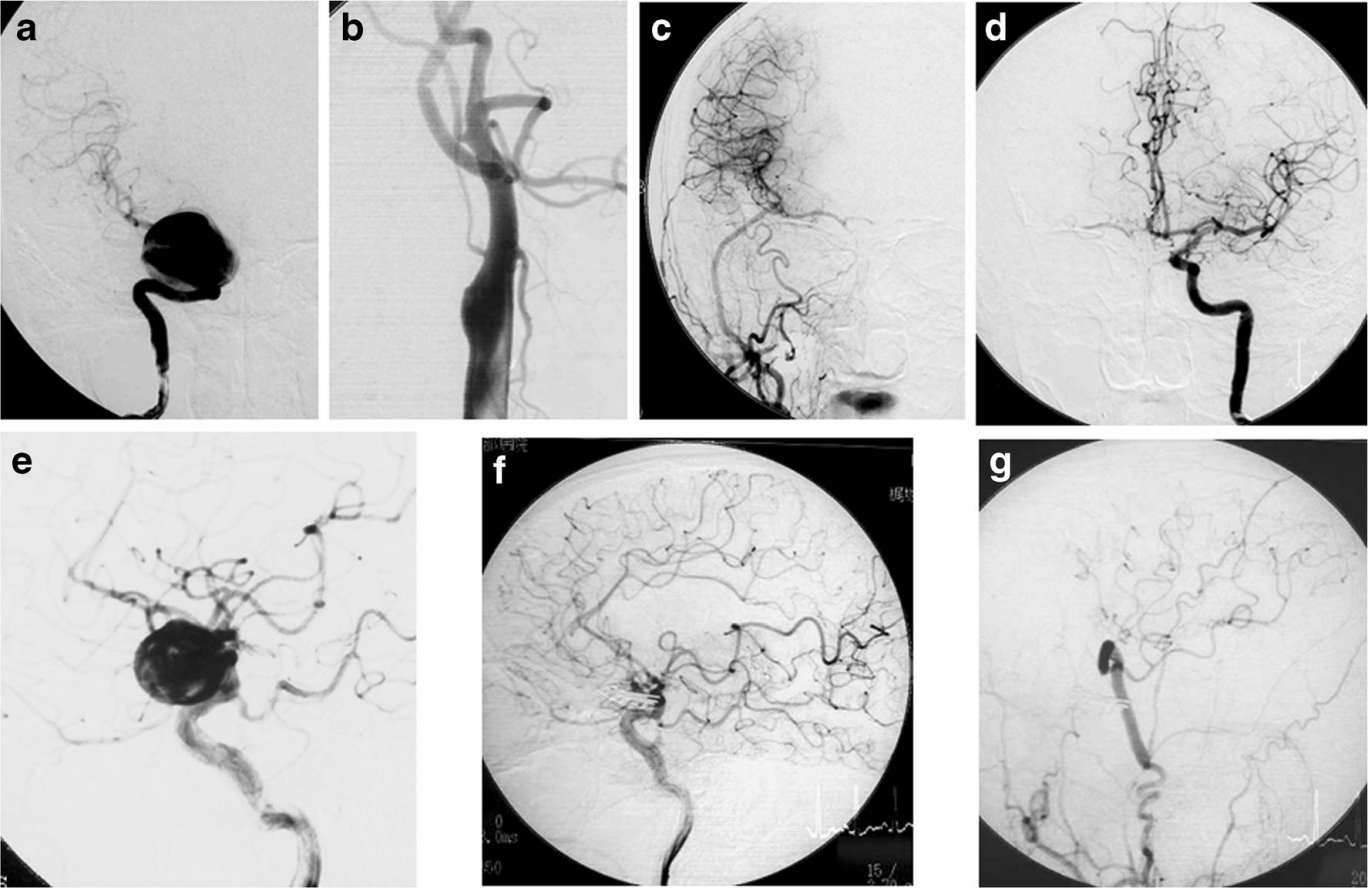
Angiographic findings. **a**–**d** A woman with a giant cavernous sinus aneurysm. She suffered from right-sided visual impairment, abducent nerve palsy, and ptosis. **a** Preoperative right internal carotid angiogram, anteroposterior view. **b**, **c** Follow-up right common carotid angiograms showing occlusion of the ICA at its origin on the lateral view (**b**) and the flow from the external carotid artery via the graft of the external carotid artery-M2 bypass intracranially reaching the distal side of the carotid bifurcation on the anteroposterior view (**c**). **d** The follow-up left internal carotid angiogram shows the cross flow of the right A1 and M1 via the anterior communicating artery on the anteroposterior view. **e**–**g** A man with a giant paraclinoid aneurysm extending anterolaterally. **e** Preoperative right internal carotid angiogram, lateral view. **f** Postoperative internal carotid angiogram showing disappearance of the aneurysm and well demonstrated flow in the ICA after complete multiple clipping. The branches of the ICA (ophthalmic, posterior communicating and anterior choroidal arteries) were preserved. **g** Postoperative external carotid angiogram showed the flow of short saphenous vein graft from STA to MCA M2 (MFB)

We have treated 16 intracavernous LGAs by ICA ligation just distal to its origin and a bypass. The clinical data and outcomes are shown in Table 1. The mean follow-up period was 102.6 ± 32.7 months (range 22–144 months). Among the 11 cases of giant and 5 cases of large aneurysms, 10 required HFB, one required MFB, and 5 required LFB. There were no MEP changes in any of the cases during the intraoperative carotid occlusion test. After confirming that, permanent proximal ligation was done by 2–0 silk. We followed up cavernous ICA aneurysms by MR imaging (Fig. 5), angiography (Fig. 6a–d) and CT. All 16 cases did not show any residual aneurysm visualization after treatment and no ischemic complications were reported. All patients of this group were discharged without any complications, except one who suffered from neck wound infection. Postoperative patency of anastomoses was confirmed in 14 patients (87.5 % of cases). Regarding the already existing cranial nerve deficit, trigeminal pain improved in all cases (5 out of 5, 100 %) and external ophthalmoplegia improved in half of the cases (5 out of 10, 50 %). Visual disturbances did not change in one patient, neither the hypoglossal nerve palsy during neck dissection in another one.

### Paraclinoid LGA cases

**Fig. 7:**
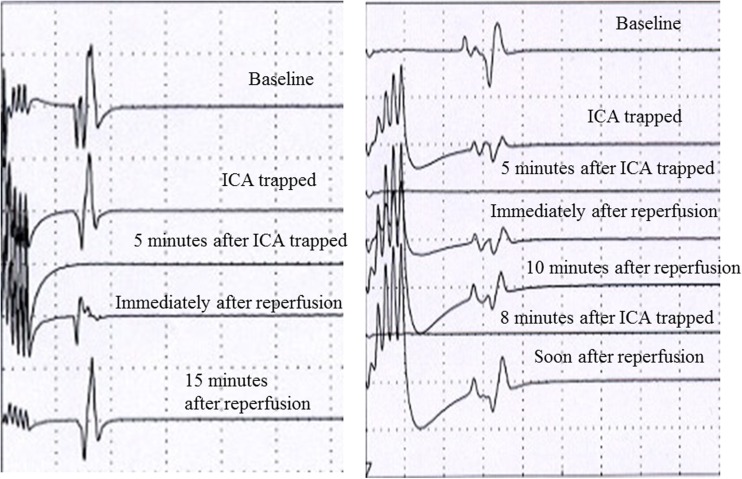
Representative MEP records demonstrating electrophysiological changes caused by trapping of the ICA. *Left* MEP disappeared 5 min after ICA trapping, however, immediately after reperfusion, the potential started recovering, becoming later the same as control. *Right* MEPs demonstrating electrophysiological changes during the SDT procedure to treat a giant paraclinoid aneurysm, requiring repeated ICA trapping and reperfusion. Initially 5 and later 8 min of trapping were used. The disappearance and recovery after the initial trapping permitted a second trapping followed by recovery at reperfusion. The procedure permitted the aneurysm to be excluded completely from circulation

Patients with paraclinoid LGAs had bypass in 13 of the 17 cases: 3 had HFB, 6 had MFB and 4 had LFB. Fifteen patients had direct clipping under SDT except one and another 2 patients, who were found unsuitable for SDT because of being a recurrence, had ICA (C2) proximal clipping with HFB. A typical case of direct clipping and MFB in this group is illustrated in Fig. 6e–g. The mean follow-up period was 79.7 ± 46.0 months (range 13–138 months). The outcome in 16 of the 17 patients showed good recovery on the Glasgow Outcome Scale (GOS) without any major ischemic complication (Table 2). Only one patient (1/17) with recurrent large aneurysm suffered large area infarction in the ipsilateral hemisphere, but was lost for long-term follow up. Significant MEP amplitude decreases lasted from 3.5 to 6 min (4.95 ± 0.7 min, *n* = 10), the longest one in a case of anterior choroidal artery trapping for SDT, but always returned to normal after reperfusion. All cases had the MEP response recovered after definitive aneurysm clipping. A representative MEP trace is shown in Fig. 7.

**Table 2:** Paraclinoid LGA cases

Case No.	Age/ Sex	An.	Angiographic assessment	BOT	Bypass graft	SDT	MEP	Treatment	GOS
1	73/F	Large An. (SAH)	Insufficient	Positive	RA-MCA (temporary)	No	N/A	Neck clipping	GR
2	53/F	Large An.	Suboptimal	N/A	Short SV	Yes	5 min	Neck clipping	GR
3	64/M	Giant An.	Suboptimal	N/A	Short SV	Yes	5 min	Neck clipping	GR
4	58/F	Large An.	Suboptimal	N/A	Short SV	Yes	5 min	Neck clipping	GR
5	54/F	Large An.	Suboptimal	N/A	Short SV	Yes	5 min	Neck clipping	GR
6	45/F	Large An.	Suboptimal	Negative	Short SV	Yes	N/A	Neck clipping	GR
7	56/F	Large An.	Optimal	N/A	No	Yes	6 min	Neck clipping	GR
8	67/F	Giant An.	Optimal	N/A	No	Yes	6 min	Neck clipping	GR
9	46/F	Large An. (SAH)	Suboptimal	N/A	Short SV	Yes	4.5 min	Neck clipping	GR
10	64/F	Giant An.	Optimal	Negative	No	Yes	4.5 min	Neck clipping	GR
11	47/F	Giant An.	Optimal	N/A	STA	Yes	No change	Neck clipping	GR
12	56/F	Large An.	Optimal	Negative	STA	Yes	No change	Neck clipping	GR
13	70/F	Large An.	Optimal	Negative	No	Yes	5 min	Neck clipping	GR
14	68/F	Large An.	Optimal	Negative	STA	Yes	3.5 min	Neck clipping	GR
15	63/M	Large An. (SAH)	Optimal	Negative	STA	Yes	No change	Neck clipping	GR
16	61/F	Large An. (recurrence)	Insufficient	Negative	High flow	No	No change	ICA clipping (C2)	SD
17	66/M	Large An. (recurrence)	Insufficient	Positive	High flow	No	No change	ICA clipping (C2)	GR

*An.* Aneurysm, *BOT* Balloon occlusion test, *GOS* Glasgow outcome scale, *GR* Good recovery, *ICA* Internal carotid artery, *LGA* Large and giant aneurysm, *MCA* Middle cerebral artery, *MEP* Motor evoked potential, *N/A* Not available, *RA* Radial artery, *SAH* Subarachnoid hemorrhage, *SD* Severe disability, *SDT* Suction-decompression technique, *STA* Superficial temporal artery, *SV* Saphenous vein

Postoperatively the occlusion of all aneurysms was confirmed by MR imaging, MR angiography, angiography, or three-dimensional CT angiography. Except for two cases with ipsilateral preoperative permanent blindness, visual disturbances showed slight improvement in three out of six patients, the rest remaining unchanged. Another two cases with cognitive dysfunction due to hydrocephalus improved spontaneously.

### Peripheral LGA cases

**Fig. 8:**
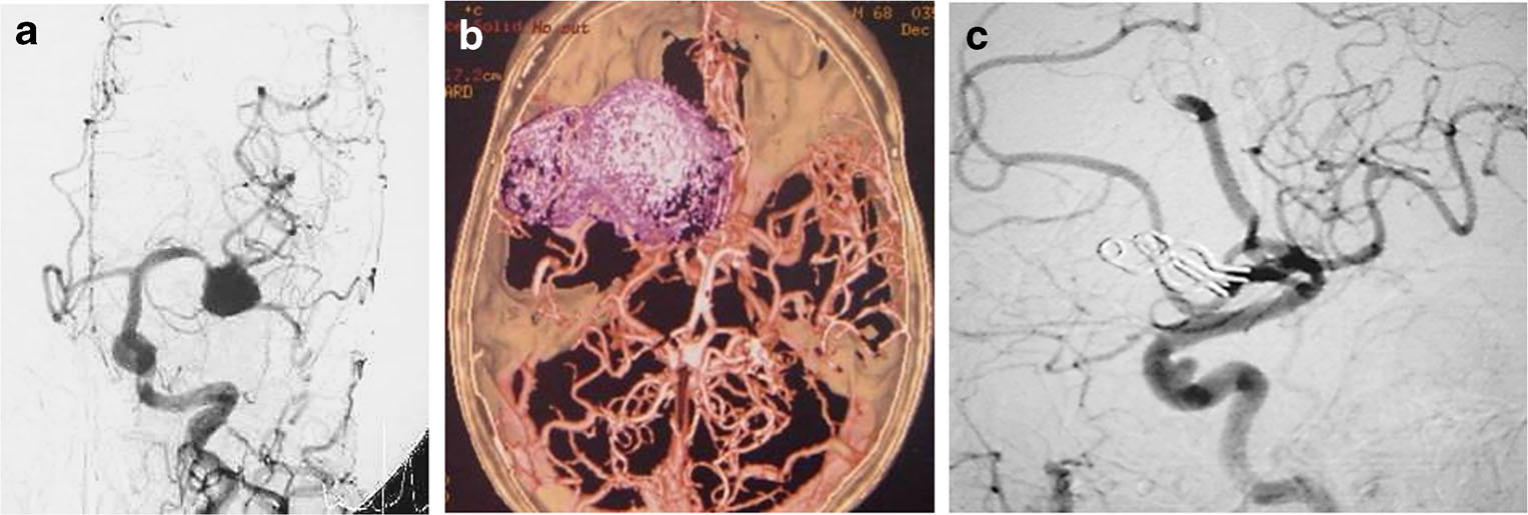
A woman with a giant thrombosed MCA aneurysm. She suffered a subarachnoid hemorrhage. **a** Left common carotid angiogram showed an irregularly shaped aneurysm of the left MCA. **b** The three-dimensional CT angiogram reveals the space occupied by the aneurysm. **c** Postoperative angiogram of lateral view showing complete disappearance of the MCA aneurysm after multi-clip occlusion, and intact M1, M2, and the flow from STA to M2 via short saphenous vein graft

Of nine peripheral LGAs, all with significant vascular wall changes, seven MCA LGAs had reconstructive clipping (*n* = 4) or trapping (*n* = 3) with bypass including LFB in three cases, MFB in one, and HFB in one. Two ACA LGAs had clipping (*n* = 1) or trapping (*n* = 1) with A3-A3 bypass. The follow-up period for all LGAs cases was 73.4 ± 37.9 months (range 15–131 months). The outcome of all 9 patients showed good GOS without any major ischemic complications (Table 3). In MCA aneurysms, complete loss or reduction to less than 50 % of MEP amplitude was observed for a minimum of 2 and a maximum of 37 min (*n* = 4), the maximum being in a case of inadvertently remained trapped lateral lenticulostriate artery origin. However, loss was reversible after release and did not influence patient’s good outcome. An illustrative case of a successfully clipped giant thrombosed MCA aneurysm preserving normal MCA flow supported with a bypass under MEP monitoring is given in Fig. 8.

**Table 3:** Peripheral LGA cases

Case No.	Age/Sex	An.	Bypass graft	Parent artery occlusion (during surgical procedure)	MEP	Treatment	GOS
1	68/M	Giant thrombosed MCA An.	Short SV	Trapping	37 min	Neck clipping	GR
2	61/F	Large thrombosed MCA An.	No	Trapping	5 min	Neck clipping	GR
3	38/F	Large thrombosed MCA An.	STA	Trapping	No change	Trapping	GR
4	57/M	Giant thrombosed MCA An.	STA	Trapping	2 min	Trapping	GR
5	61/F	Giant thrombosed MCA An.	STA	Trapping	7 min	Neck clipping	GR
6	58/F	Giant thrombosed MCA An.	No	No	No change	Neck clipping	GR
7	32/M	Large fusiform MCA An.	RA	Trapping	No change	Trapping	GR
8	65/F	Thrombosed A2 An. (SAH)	A3-A3	No	No change	Neck clipping	GR
9	42/M	Dissecting A2 An. (SAH)	A3-A3	Trapping	No change	Trapping	GR

*An.* Aneurysm, *GOS* Glasgow outcome scale, *GR* Good recovery, *LGA* Large and giant aneurysm, *MCA* Middle cerebral artery, *MEP* Motor evoked potential, *SAH* Subarachnoid hemorrhage, *STA* Superficial temporal artery, *SV* Saphenous vein, *RA* Radial artery

## Discussion

In recent years, the attention to the management of LGAs has been increasing. However, the majority of published reports reflect the outcome of specific for a single institution series [2], a uniform team strategy [13], or present small series and case reports [16]. Thus, the principal objective of this study was to analyze the outcome of LGA management under a specific protocol focused on *overprotection* from ischemia, and at the same time allowing adaptive flexibility according to the expected ischemia tolerance. The amount of the necessary blood flow to avoid ischemic complications in both cortex and deep white and gray matter, supplied via major arterial trunks and their respective perforating arteries has been subject to multiple studies, applying different methodologies of CBF evaluation without definitive conclusion. Based on the existing information around the year 2000, we attempted a simple protocol, addressing the major pitfalls of this surgery at that time. In the context of the current status of LGA management, the obtained experience confirms that being *overprotective* by providing generous bypass flow and performing frequent reperfusions during trapping under monitoring in these patients can yield results not inferior than those involving sophisticated and detailed CBF evaluations.

Recently published studies have used two major groups of methods: open [10, 15, 22, 24, 26] and endovascular [7, 11, 17]. Endovascular techniques unfortunately did not show in LGAs the promising results obtained in small aneurysms. Recent stent and flow-diverting techniques have indicated rates of recurrence and complications that cannot be neglected. Therefore, open surgical techniques continued being applied widely in experienced centers around the world. However, even in big centers, the series are not sufficiently big and differ significantly in patient selection and the applied diagnostic and surgical technology to permit conclusions of high evidence level [2, 14]. In some of them, unfortunately the criteria were changing through the series [2, 6], which makes the comparison among studies difficult.

One of the most important components of a LGA management protocol is the ischemia tolerance evaluation and ischemia protection. Many studies [2, 5, 14] have used algorithms addressing ischemia protection as *tolerating or not ICA occlusion* (in need or not of HFB), often combined with the results of diagnostic CBF measurements [4, 17, 24], or applied standardized protocols of a *uniform* surgical technique [13]. While bypass revascularization addressed to some extent flow compensation and augmentation in the main trunks, the major difficulties of treatment of complex LGA of the carotid circulation remain with perforating arteries flow preservation during parent artery occlusion [33]. We believe that each of these problems should be addressed separately and has different characteristics and impact in the three groups of aneurysms in our study.

In intracavernous LGAs, evaluation addressed the existing uncertainties in preoperative testing before permanent carotid occlusion. BOT has been a mainstay in carotid occlusion testing for nearly four decades, since Serbinenko described its first clinical application in 1974 [23]. Therefore, we adopted the clinical BOT for the patients under consideration for ICA occlusion. However, even in clinical BOT *permitting ICA occlusion* patients, incidence of ischemic stroke was only reduced, but not eliminated. Improvements were attempted with additional flow evaluation methods [27], with added hypotensive challenge, but without definitive solution of the problem. Attempting to simplify the approach to the problem on the background of *overprotective* bypass supply, we evaluated conventional angiography flow (with Matas and Alcock tests) and classified the collateralization of arterial territories and timing of flow as *insufficient, suboptimal*, and *sufficient*. The differentiation between *suboptimal* and *sufficient* was chosen to be 2 s or more delay in the venous phase between injected and occluded hemispheres as a clear to define margin on visually assessed serial angiographies. Using values of less than 2 s was considered prone to misinterpretation of angiography timing or influenced by physiological variation. Then, we have decided 2 s as the first clear landmark of venous delay. At the time of introducing this criterion, there were no established standards of venous delay. We adopted these criteria after the role of venous return delay in assessing collateralization was mentioned as a possible useful parameter [18]. In a later than the beginning of our study publication, the value of venous phase delay has been emphasized by van Rooij et al. [30]. The surgical strategy of overprotection was based on the performance of a bypass presumably providing flow in excess of the deficit expected. For that in cases of *insufficient* collaterals we always chose the HFB, in the *suboptimal* category we chose the MFB, and even in cases of *optimal* collateralization, where protective revascularization might not be needed, we chose the LFB unless technically unfeasible. A *second line of protection* in this group was the intraoperative monitoring of MEPs. There were no intraoperative changes during the temporary occlusion in all cases of this group, confirming that the preoperative choice of bypass was adequate.

In the paraclinoid LGA group, the two distinctive risks of ischemia due to main trunk and perforators occlusion needed separate approach. Aneurysm volume reduction allowed faster repositioning of trapping clips with shorter flow interruptions and delay of the intentional puncture of the aneurysm for thrombectomy as much as possible with the help of SDT. All the applied surgical techniques required ischemia tolerance evaluation as they required also at least temporary arterial occlusion, so the patients were evaluated angiographically for ICA occlusion with the same criteria as the intracavernous LGA group, with BOT and selection of bypass under the same rules. Only in two cases of recurrent LGA, where intracranial dissection and clipping were considered not possible without unreasonably high risk, proximal occlusion was performed under the biggest available ischemia protection—HFB.

Intraoperative ischemia protection for the deep gray and white matter, particularly the corticospinal tract in this group was based on MEP monitoring. MEPs had broad application for the last decade, including even cortico-bulbar potentials [33]. For the terminal perforating arteries arising from the aneurysm portions that had to be occluded, we do not have yet an established a broadly acceptable management strategy. Direct revascularization of perforators is not yet possible. Therefore, all that we can do for the deep gray and white matter was to evaluate by MEP monitoring the induced degree of ischemia on the corticospinal tract in the internal capsule, also a territory of the anterior choroidal artery. During parent vessel trapping, if the insufficient retrograde flow from distal ICA and posterior communicating arteries lead to hypoperfusion, MEP changes served as an early warning, indicating a stage of reversible *penumbra* [29]. Even if the blood flow reduction in these circumstances reached the point of electrophysiological silence, resulting from loss of membrane ion gradients and energy metabolites [6], the metabolic energy reserves would have allowed structural preservation. Continued trapping probably for 15 min or more would have exhausted this limited capacity and transformed the penumbra zone into irreversibly damaged tissue [29]. From the results in our 10 cases with MEP changes during temporary trapping, the interval from trapping to the onset of ischemic penumbra was 3.5–6 min, with an average of approximately 5 min (Fig. 7). We used clip repositioning for reperfusion at the appearance of MEP changes. This time seems to be consistent with the results of our previous research on mini pig model with anterior choroidal artery occlusion, where the time of penumbra onset in anterior choroidal artery occlusion was 6 min and reperfusion within less than 15 min salvaged the penumbra area without any permanent ischemic lesion, even though MEPs had already disappeared [29]. Since we could expect safe recovery within the above-mentioned or maybe a little longer time duration, under MEP monitoring we could avoid the ischemic complications associated with perforating arteries in this series. The exact range of tolerable occlusion time in these patients has still to be established.

The peripheral thrombosed LGA group was evaluated for ischemia protection only by angiography. A3 to A3 anastomoses were considered well matching the existing flow demand as a LFB for the two ACA LGAs. MCA LGAs required attention for eventual relationship of lenticulostriate arteries to dissection and clip placement. In all of these cases except one, MEP changes occurred before 10 min of flow interruption, in a similar way as the paraclinoid LGA, and required management by clip repositioning. One inadvertent occlusion of a lenticulostriate artery led to prolonged MEP changes for 37 min not resulting in any neurological damage, indicating the existence of occasional unpredictable flow reserves. We did not encounter aneurysmal necks involving the MCA bifurcation which would have required more complex reconstructions and eventually multiple anastomoses.

The outcome of our patients is indicating an effective strategy of management. All three groups had good recovery for all patients, except one in the paraclinoid LGA group, who developed severe ischemic stroke after surgery for a recurrent large aneurysm, in spite of the patent HFB. Complications review showed only few local surgical complications: surgical site infection at the neck in one patient and one iatrogenic hypoglossal nerve damage during neck dissection.

This study had the limitations of being a single center, retrospective non-randomized cohort, and lacked the inclusion of the whole range of randomly treated LGA lesions, resulting from the bias of referral to our institution. The absence of C1 as well as ICA and MCA bifurcation lesions may have had a positive impact on the total outcome, but it allowed better standardization of the groups. Overall, the validity of our observations should be applied to the groups of LGAs included in this study.

## Conclusions

Ischemia *overprotection* protocol has shown to be effective in the management of LGAs with the locations and types in our series, yielding excellent results and low complication rates. Without quantitative estimation, bypass oversupply of the hemisphere appears to be protective to clinically detectable ischemia without clinical indications of hyperperfusion. Whether the angiographic evaluation is sufficient for this protocol can be answered in the future with comparable results based on different evaluation methods, criteria, and types of lesions.
